# Multitargeted biological actions of polydatin in preventing pseudogout acute attack

**DOI:** 10.3389/fmolb.2025.1553912

**Published:** 2025-02-27

**Authors:** Chiara Baggio, Paola Galozzi, Amelia Damasco, Vanni Lazzarin, Giampietro Ravagnan, Paolo Sfriso, Roberta Ramonda, Leonardo Punzi, Gianmaria Pennelli, Andrea Doria, Roberto Luisetto, Francesca Oliviero

**Affiliations:** ^1^ Rheumatology Unit, Department of Medicine-DIMED, University of Padova, Padova, Italy; ^2^ Laboratory Medicine Unit, Department of Medicine DIMED, University of Padova, Padova, Italy; ^3^ Surgical Pathology Unit, Department of Medicine-DIMED, University of Padova, Padova, Italy; ^4^ Institute of Translational Pharmacology-National Research Council, Rome, Italy; ^5^ Centre for Gout and Metabolic Bone and Joint Diseases, SS Giovanni and Paolo Hospital, Venice, Italy; ^6^ Department of Surgery, Oncology and Gastroenterology-DISCOG, University of Padova, Padova, Italy

**Keywords:** polydatin, calcium pyrophosphate crystals, crystal-induced inflammation, inflammation, interleukin-1β

## Abstract

**Introduction:**

We have recently shown that polydatin (PD) prevents calcium pyrophosphate (CPP) crystal-induced arthritis in mice. This study aims to explore potential mechanisms of action associated with this anti-inflammatory effect.

**Materials and methods:**

Acute arthritis was induced in Balb/c mice by the injection of crystals into the ankle joint. Animals were randomised to receive PD or colchicine according to a prophylactic protocol. Ankle swelling was measured and both joints and muscles were harvested at sacrifice. Histological evaluations were performed using H&E staining to assess cartilage and muscle damage. Kondziela’s inverted test was used to assess muscle strength. An exploratory protein array was performed on joint tissue to identify relevant inflammatory pathways. Human monocytes pretreated with PD were stimulated with CPP crystals. The use of specific inhibitors was instrumental in demonstrating their anti-inflammatory effects and assessing the role of SIRT1. The chemotaxis assay was performed to test the effect of PD and J-113863 on PBMCs migration in response to plasma and synovial fluids. Cytokine levels were measured by ELISA.

**Results:**

CPP crystals injection resulted in swelling, leukocyte infiltration, loss of synovial membrane structure homogeneity. Mice pretreated with PD showed reduced ankle swelling and this was associated with very limited inflammatory damage. Regarding the effect on gastrocnemius muscle, crystals induced leukocyte infiltration and edema. PD and colchicine treatment reduced muscle damage and preserved musculoskeletal structure in mice. The cytokine array revealed the activation of various inflammatory pathways after CPP injection and PD was shown to influence leukocyte migration, angiogenesis and inflammation. *In vitro*, PD reduced inflammatory cytokines, chemokines and VEGF levels. CCR-1 inhibition was effective in reducing pro-inflammatory mediator levels in CPP treated monocytes and in reducing PBMCs migration. The anti-inflammatory action of PD also involved SIRT-1 activation, and its inhibition reverted the beneficial effects of PD. Finally, PD reduced the PBMCs migration in response to synovial fluids.

**Conclusion:**

PD effectively prevents inflammatory responses to CPP crystals in mice, preserving both articular and muscular structures. Its anti-inflammatory effects are primarily mediated through pathways regulating leukocyte migration and the suppression of pro-inflammatory mediators.

## Graphical Abstract

**Figure:**
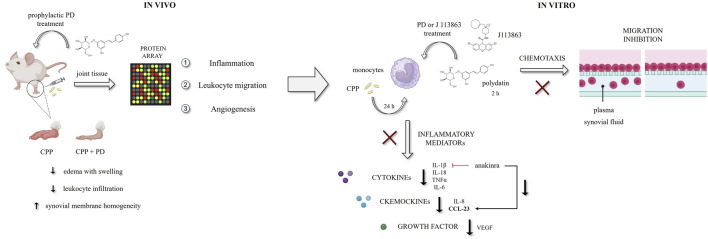

## 1 Introduction

Calcium pyrophosphate crystal deposition (CPPD) in the articular and periarticular tissues causes acute or chronic inflammatory arthritis. CPP crystals trigger the activation of the NLRP3 inflammasome and subsequently, the release of cytokines and other pro-inflammatory mediators (chemokines, reactive oxygen species) from the inflammatory cell infiltrate (Pascart et al., 2024; Oliviero et al., 2020; Zamudio-Cuevas et al., 2015; Martinon et al., 2006).

Clinically, CPP crystal arthritis is characterised by swelling, pain, limited joint function, and, over time, tissue damage and disability (Mahon and Dunne, 2018).

Unlike gout, where monosodium urate (MSU) crystal deposition can be prevented with hypouricemic drugs, no available treatments can effectively prevent CPP crystal formation or promote their dissolution (Andrés et al., 2018; Abhishek et al., 2018). As a consequence, the management of CPPD focuses on treating inflammation with oral non-steroidal anti-inflammatory drugs (NSAIDs) and colchicine, and corticosteroids administered both by intra-articular injections and orally (Stamp et al., 2018; Zhang et al., 2011; Latourte et al., 2024).

In recent years, there has been an increase in research regarding the health benefits attributed to polyphenols. These natural compounds have shown a wide range of favorable effects in many inflammatory chronic conditions (Billingsley and Carbone, 2018; Ding et al., 2018; Oliviero et al., 2018; Cory et al., 2018).

Polydatin (3,5,4-trihydroxystilbene-3-O-beta-D-glucopyranoside, PD) is a potent natural stilbenoid polyphenol and a glycoside precursor of resveratrol with improved bioavailability. It is mainly contained in grapes and the bark of Polygonum cuspidate. Recent studies suggest that PD exerts its effects by modulating signaling pathways involved in inflammation, oxidative stress, and apoptosis (Tang et al., 2019; Lv et al., 2018; Yang et al., 2019; Ravagnan et al., 2013). While the pharmacological mechanisms of PD are still not fully elucidated, it has been postulated that PD acts through the activation of SIRT1 (Sun et al., 2021). Chen et al. showed that PD inhibit NF-κB/NLRP3 inflammasome activation via the AMPK/SIRT1 pathway (Chen and Lan, 2017). In addition, PD has been found to have significant protective effects on diseases associated with oxidative stress by regulating SIRT1-related targets (Liu et al., 2024). However, only a few studies have evaluated the role of PD in crystal-induced inflammation and especially its effect on CPP crystal-induced arthritis (Du et al., 2023; Oliviero et al., 2019; Oliviero et al., 2021).

Previously, we have shown that PD is able to prevent the inflammatory response in an *in vitro* and *in vivo* model of acute CPP-induced inflammation and arthritis. In particular, PD strongly inhibited IL-1β release from THP-1 stimulated by CPP crystals and ROS, NO production *in vitro*. *In vivo*, prophylactic treatment with PD significantly reduced ankle swelling, leukocyte infiltration, necrosis, edema, and synovitis after 48 h from crystal injection. PD also affected the circulating levels of IL-1β and CXCL1 and their tissue expression (Oliviero et al., 2019; Oliviero et al., 2021).

Therefore, in this study we aimed at evaluating the effect of PD in a mouse model of acute arthritis induced by CPP crystal injection in the ankle. Additionally, we conducted *in vitro* experiments to explore the mechanisms underlying PD anti-inflammatory effects. In particular, we focused on specific pathways overexpressed in the joints of mice following treatment with crystals, including leukocyte migration, proinflammatory mediators and angiogenesis.

## 2 Materials and methods

### 2.1 *In vivo* experiments

#### 2.1.1 Mice

Male, wild type, Balb/c mice of 10 weeks of age were bred and maintained under specific pathogen-free conditions at the animal facility of the Interdepartmental Research Center of the Experimental Surgery of Padova University. All animal care and experimentation were conducted in compliance with the guide lines of the European Union Directive 2010/63 and the Italian Law D.Lgs. 26/2014 and with the approval of the Institutional Animal Experimentation Ethics Committee of Padova University and the Italian Health Ministry (Rome, Italy) registered under #102/2020-PR.

#### 2.1.2 CPP crystal-induced arthritis development

Acute arthritis was induced by the injection of a suspension of 0.3 mg sterile CPP crystals (InvivoGen, Aurogene, Italy) in 20 µL PBS into the right ankle joint of the mice. Injections were performed under inhalant anesthesia (Sevorane®, Abbott, 4% induction, 1.5% maintenance) using a Fluovac respiratory system (Harvard Apparatus, Holliston, MA, USA) and microliter syringes #705 (Hamilton, Reno, USA) with 27 G beveled needles. Animals were randomized into four groups (n = 5 per group) receiving: (1) i.a. PBS (control group), (2) i.a. CPP crystals, (3) i.a. CPP crystals + PD, (4) i.a. CPP crystals + colchicine (control drug). Ankle swelling was measured at different time points using a precision digital caliper (Kroeplin Gmbh, Schlüchtern, Germany). To avoid any attribution bias, ankle swelling was measured by an investigator who was blinded to the group allocation. Forty-8 hours after the injection of CPP crystals (peak of the acute phase) mice were euthanized, and ankle joints and gastrocnemius were collected for inflammatory mediators’ assessment and histological analysis (Oliviero et al., 2021).

#### 2.1.3 Drugs

PD was extracted from Polygonum cuspidatum, according to the procedure described in patent EP 1292320 B1; and kindly supplied by GLURES Srl (a spin-off of the National Research Council, Rome, Italy, purity >99%). Colchicine was obtained from Sigma-Aldrich (Milan, Italy).

#### 2.1.4 Treatment with PD and colchicine

PD and colchicine were administered by gavage at 40 mg/kg and 1 mg/kg in 200 LPBS/EtOH/glucose, respectively, according to a prophylactic protocol: 24, 15 and 1 h before and 1, 6 and 24 h after i.a. injection of CPP crystals. These time points were chosen based on previous experimental data obtained by us and others (Oliviero et al., 2021; Reber et al., 2017).

#### 2.1.5 Histological assessment

Histopathological evaluations were conducted on five mice per arm. The ankle joints were dissected, fixed in 10% buffered formalin, decalcified for 24 h using a solution of formic and nitric acid, embedded in paraffin and sectioned at 4-μm. Tissue sections were stained with hematoxylin and eosin (H&E) for evaluation (Hayer et al., 2021). The degree of synovial inflammation and bone erosions were scored according to the Standardised Microscopic Arthritis Scoring of Histological sections (‘SMASH’) recommendations as follows: 0, normal; 1, mild; 2, moderate; 3, severe (Hayer et al., 2021). Dissected gastrocnemius muscles were fixed in 10% formalin, embedded in paraffin, cut into 4-μm thick sections and stained with H&E for morphological analysis to assess changes in muscle morphology after CPP crystals injection. The slides were examined semi-quantitatively using a modified scale ranging from 0 to 3 to evaluate edema and inflammation (Kondziella, 1964). All sections were analyzed using a Leica DM4000B microscope provided with a Leica DFC420 camera.

#### 2.1.6 Kondziela’s inverted test

The Kondziela’s inverted test has been performed according to the standard protocol (Kondziella, 1964) to assess the grip strength of the mouse’s hindlimbs and forelimbs. The test was executed using a mesh screen consisting of 12 squares of 1 mm diameter wire. The mouse was placed at the center of the screen and inverted to check the grip of the mouse’s limbs. The time (sec) of the fall was noted with a cut-off time of 60 s after which the mouse was removed. The scoring was attributed as follow: falling between 1–10 s = 1, 11–25 s = 2, 26–60 s = 3, after 60 s = 4 (Oliviero et al., 2021).

#### 2.1.7 Protein array

Cytokine profiling was examined on frozen murine articular tissue using the Mouse Cytokine Array C1000 kit (RayBiotech, Inc.). Tissues were first homogenized to extract and quantify proteins. Lysates from two ankles per condition were combined and processed according to the manufacturer’s protocol. For each sample, the dot intensity on each membrane were analyzed using the ImageJ software, enabling a quantitative evaluation of signal intensity. The detected signals were normalized to the positive control and corrected by subtracting the background signal.

### 2.2 *In vitro* experiments

#### 2.2.1 PBMCs and monocytes isolation

Buffy coat from healthy donors (HDs) (n = 4) was used for *in vitro* assay. Peripheral blood mononuclear cells (PBMCs) were isolated by density-gradient centrifugation using Histopaque 1,077 (Sigma-Aldrich). PBMCs were seeded into 96-well plates at 2.5 × 10^5^ cells/well, in RPMI 1640 (Sigma-Aldrich) supplemented with 10% fetal bovine serum (FBS, Sigma-Aldrich) and with 1% glutamine (Sigma-Aldrich) and 1% penicillin-streptomycin (Sigma-Aldrich) (complete medium). Monocytes were isolated from mononuclear cells by exploiting their ability to adhere to plastic. The next day, the supernatant was removed and replaced with RPMI complete medium.

#### 2.2.2 *In vitro* assay

For the *in vitro* studies, monocytes were stimulated with sterile CPP (final concentration 0.025 mg/mL) for 24 h in RPMI 1640 supplemented with 2.5% FBS, 1% glutamine and 1% penicillin-streptomycin. Where indicated, polydatin (100 µM) was added 2 h before the stimulation with crystals and anakinra (0.1 µg/mL), J113863 (Medchemexpress, Monmouth Junction, USA, 10 µM) and EX527 (Selleckchem, Cologne, 10 µM) were added 30 min before the use of polydatin. Cells incubated with medium alone served as controls. All experiments were performed three-four independent times.

#### 2.2.3 Synovial fluid collection

Synovial fluid (SF) was collected by arthrocentesis from swollen knees of untreated patients with acute CPP crystal-induced arthritis attending the outpatients’ clinic of the Rheumatology Unit of the University of Padova. Four discarded samples of SFs were tested in this study. SFs characteristics were reported in Supplementary Table S1. The SF was examined as part of routine procedures and examination including ordinary light microscopy for total and differential white blood cell (WBC) count. Differential WBC provided the percentage of polymorphonuclear (PMN), monocytes (M) in the SF. Crystal search was performed using polarized compensated light microscopy. After examination, SF samples collected in EDTA were centrifuged at 1,500 rpm for 30 min to remove the cells, particulate material, and debris. SF samples were obtained and studied under protocol that included written informed consent and that was approved by the local Institutional Review Board (approval #39872).

#### 2.2.4 ELISA assay

Cell culture supernatants from different experimental conditions were examined for IL-1β (Thermofisher Scientific, Massachusetts, USA; sensitivity: 2 pg/mL), IL-18 (R&D Systems, Minneapolis, USA; sensitivity: 5.5 pg/mL), IL-6 (Thermofisher Scientific; sensitivity: 2 pg/mL), TNFα (BioLegend, San Diego California, USA; sensitivity: 2 pg/mL), IL-8 (Thermofisher Scientific; sensitivity: 2 pg/mL), CCL-23 (R&D Systems; sensitivity: 2 pg/mL) and VEGF (BioLegend; sensitivity: 4 pg/mL) concentration commercially available enzyme-linked immunosorbent assay (ELISA) kits. Synovial fluid CCL-23 concentration was also assessed by ELISA (dilution: 1:2 in PBS).

#### 2.2.5 Chemotaxis assay

Chemotaxis experiments were performed in a 48-well modified Boyden chamber (Neuro Probe, Gaithersburg, MD) using 5 µm nucleo-pore polyvinyl pyrrolidine-free polycarbonate filters. As chemotactic stimuli, SFs from patients with pyrophosphate crystal-induced arthritis were added to lower chambers (30 µL) after appropriate dilution (5% in RPMI 0% FBS supplemented with 1% penicillin-streptomycin). RPMI plus 5% plasma from heathy donor was used as a positive migration control, while RPMI plus 1% FBS was used as a negative control. Upper chambers were filled with 50 μL PBMCs suspension (50,000 cells/50 μL in RPMI with 1% FBS and 1% penicillin) in the presence or absence of polydatin (overnight treatment, 100µM and 200 µM) or J113863 (10 µM) as indicated. After 1.30 h incubation at 37°C, non-migrating cells on the upper filter surface were removed by scraping. The cells that had migrated to the lower side of the filter were stained with Diff Quik staining, and densitometry was performed using ImageJ. Each condition was performed in sextuplicate. Results are reported as optical density (OD) arbitrary units.

### 2.3 Statistical analysis

All experiments were performed in at least three independent replicates. The Shapiro-Wilk test was used to analyse the distribution of continuous variables, and variables with a normal distribution were presented as media ±standard deviation (SD). For normally distributed data, Anova test followed by Bonferroni’s multiple comparisons test was used for multiple comparisons. Intra-group comparisons were also evaluated by the paired-samples t-test. Statistical analysis was performed with GraphPad Prism 8 (GraphPad Software Inc., La Jolla, CA, United States). A p-value <0.05 was considered significant.

## 3 Results

### 3.1 Prophylactic oral treatment with PD prevents CPP crystal-induced arthritis in mice

**FIGURE 1:**
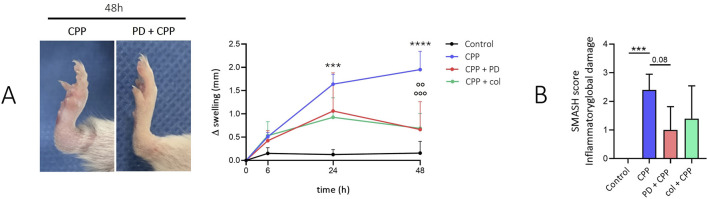
Preventive treatment with polydatin inhibits the development of CPP-induced arthritis in mice. Mice (n = 5/group) received PD (40 mg/kg) or colchicine (1 mg/kg) treatment by gavage before the i.a. injection of CPP crystals (0.3 mg/20 µL). **(A)** *Left panel*: Ankles after 48 h from CPP injection and prophylactic treatment with PD. Right panel: Variation in ankle swelling after CPP crystal injection and drug treatment in the prophylactic group. Data are expressed as mean ± SD. p calculated according to the One Way Anova, Bonferroni’s multiple comparisons test: ***p < 0.001, ****p < 0.0001 vs. control; °°°p < 0.001 vs. CPP. **(B)** SMASH score calculated for n = 5 ankle sections. Data are expressed as mean ± SD. p calculated according to the One Way Anova, Bonferroni’s multiple comparisons test: ***p < 0.001. CPP, calcium pyrophosphate crystals; PD, polydatin; col, colchicine.

Injection of CPP crystals into the ankle of the mice caused an increase in joint swelling that was maximal at 48 h (Figure 1A). Prophylactic treatment with PD significantly reduced ankle swelling after 48 h (p < 0.001), similar to that seen after colchicine pretreatment (p < 0.01). In mice treated with CPP crystals for 48 h, histological analysis revealed areas of edema, moderate areas of leukocyte infiltrate, and inhomogeneity of the synovial membrane. In mice pretreated with PD and colchicine, inflammatory damage appeared very limited with significant conservation of bone structures. In untreated joints, we observed intact bone surface. Inflammatory global damage (SMASH score) was higher in mice treated with CPP than in mice pretreated with PD (p < 0.08) (Figure 1B).

### 3.2 Prophylactic oral treatment with PD prevents crystal-induced muscle inflammatory damage and enhance muscular strength in mice

**FIGURE 2:**
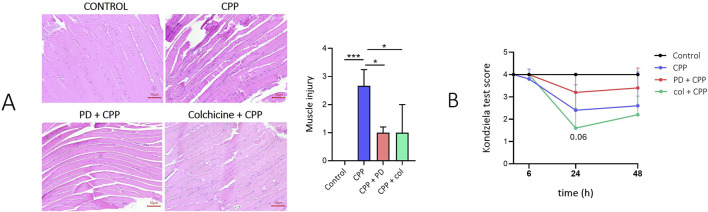
Preventive treatment with polydatin prevents muscle damage and improves muscle strength in mice treated with CPP crystals. Mice (n = 5/group) received PD (40 mg/kg) or colchicine (1 mg/kg) treatment by gavage before the i.a. injection of CPP crystals (0.3 mg/20 µL). **(A)** *Left panel*: muscle longitudinal sections show evident expansion of the interstitial spaces and leukocyte infiltration 48 h after CPP crystal injection. Edema is reduced in mice pretreated with PD and colchicine. H and E staining. *Right panel*: Muscle injury score. Data are expressed as mean ± SD. p calculated according to the One Way Anova, Bonferroni’s multiple comparisons test: *p < 0.05, ***p < 0.001. **(B)** Kondziela test score in mice 48 h after CPP crystal injection. Data are expressed as mean ± SD. p calculated according to the One Way Anova, Bonferroni’s multiple comparisons test. CPP, calcium pyrophosphate crystals; PD, polydatin; col, colchicine; H and E, hematoxylin and eosin.

In mice treated with CPP crystals for 48 h, cross sections of gastrocnemius muscles showed large number of inflammatory cell infiltration, sign of hypereosinophilic degenerative fibers and areas of expanded interstitial spaces between muscle fibres and bundles. In mice pretreated with PD and colchicine, muscle damage and edema were more limited and the musculoskeletal structure was generally conserved. Muscle injury score was higher in mice treated with CPP than in mice pretreated with PD or colchicine (p < 0.05) (Figure 2A). The Kondziela’s inverted test was used to measure muscular strength of the animals using all four limbs. Although not significant, PD pretreatment partially prevents the loss of muscle strength compared to mice that received only CPP injection (Figure 2B).

### 3.3 The effect of PD pretreatment on IL-1β, IL-18, IL-6, TNFα, IL-8, CCL-23 and VEGF levels

The protein array (pilot experiment, n = 1) showed the activation of different inflammatory pathways 24 h after CPP injection. PD was shown to strongly influence leukocyte migration (MIP-1γ, VCAM, L-selectin, BLC), angiogenesis (VEGF, VEGF-R2, VEGF-R3, VEGF-D, VEGF) and inflammatory mediators (IL-1β, IL-6, IL-1α) (Supplementary Figure S1). Based on these preliminary results, we decided to investigate *in vitro* the effect of PD on (1) the release of inflammatory mediators and VEGF, (2) the release of CCL-23, a chemochine that acts through the CCR1 receptor and with properties similar to MIP-1γ, and finally (3) the migration induced by chemotactic stimuli and synovial fluids from patients with pyrophosphate crystal-induced arthritis.

**FIGURE 3:**
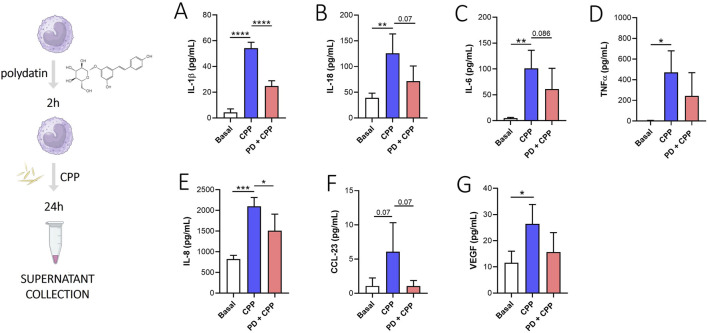
Effect of PD on CPP-stimulated IL-1β, IL-18, TNFα, IL-8, CCL-23 and VEGF production. Monocytes were pretreated with PD (100 µM) for 2 h and then stimulated for 24 h with CPP crystals (0.025 mg/mL). **(A)** IL-1β, **(B)** IL-18, **(C)** IL-6, **(D)** TNFα, **(E)** IL-8, **(F)** CCL-23 and **(G)** VEGF levels in supernatants were quantified by ELISA. Data are expressed as mean of four independent experiments ±SD. p calculated according to the One Way Anova, Bonferroni’s multiple comparisons test: *p < 0.05, **p < 0.01, ***p < 0.001, ****p < 0.0001. CPP, calcium pyrophosphate crystals; PD, polydatin.

As shown in Figure 3, monocytes treated with CPP crystals for 24 h released moderate to higher levels of IL-1β (Figure 3A, p < 0.0001), IL-18 (Figure 3B, p < 0.01), IL-6 (Figure 3C, p < 0.01), TNFα (Figure 3D, p < 0.05), IL-8 (Figure 3E, p < 0.001), CCL-23 (Figure 3F, p = 0.07) and VEGF (Figure 3G, p < 0.05) compared to basal condition. Pretreatment with PD inhibited IL-1β (p < 0.0001) and IL-8 (p < 0.05) release induced by CPP crystals after 24 h. Furthermore, although not significantly, PD also appears to reduce IL-18, IL-6, TNFα, CCL-23 and VEGF release compared to baseline. Taken together, these results showed that PD acts through modulation of different mediators: pro-inflammatory cytokines (IL-1β, IL-18, IL-6, TNFα) involved in CPP mediated inflammation, chemochines (IL-8, CCL-23) and growth factors (VEGF) involved in leukocyte migration also through the endothelial cell activation and permeabilization.

**FIGURE 4:**
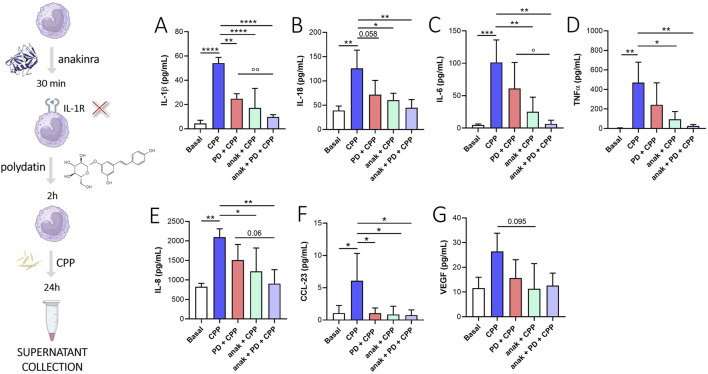
Effect of IL-1β signal inhibition on PD treatment. Monocytes were pretreated with PD (100 µM) for 2 h and then stimulated for 24 h with CPP (0.025 mg/mL). Where indicated, anakinra (anak, 0.1 µg/mL) was added 30 min before the PD treatment. **(A)** IL-1β, **(B)** IL-18, **(C)** IL-6, **(D)** TNFα, **(E)** IL-8, **(F)** CCL-23 and **(G)** VEGF levels in supernatants were quantified by ELISA. Data are expressed as mean of four independent experiments ±SD. p calculated according to the One Way Anova, Bonferroni’s multiple comparisons test: *p < 0.05, **p < 0.01, ***p < 0.001, ****p < 0.0001. p calculated according to t-test, PD + CPP vs anak + PD + CPP: °p < 0.05, °°p < 0.01. CPP, calcium pyrophosphate crystals; PD, polydatin; anak, anakinra.

To evaluate whether the effect of PD was mediated by IL-1β signaling inhibition, we used anakinra (IL-1β receptor inhibitor) in combination with PD (Figure 4). We found that anakinra + PD treatment decrease IL-1β (Figure 4A, p < 0.0001), IL-18 (Figure 4B, p < 0.01), IL-6 (Figure 4C, p < 0.01), TNFα (Figure 4D, p < 0.01), IL-8 (Figure 4E, p < 0.01) and CCL-23 (Figure 4F, p < 0.05) release from CPP crystal-stimulated monocytes after 24 h. Although not significantly, anakinra + PD treatment also appears to reduce VEGF release compared to baseline (Figure 4G). Intragroup differences showed that anakinra + PD treatment blocks the release of IL-1β (p < 0.01) and IL-6 (p < 0.05) more than PD treatment alone. Although not significant, this trend was also observed for IL-18, TNFα and IL-8, suggesting that PD, in addition to modulate IL-1β signal, acts also through other mechanisms of action.

### 3.4 CCR1 inhibition decreases the release of IL-1β, IL-18, IL-6, TNFα, IL-8 and VEGF from monocytes stimulated with CPP crystals

**FIGURE 5:**
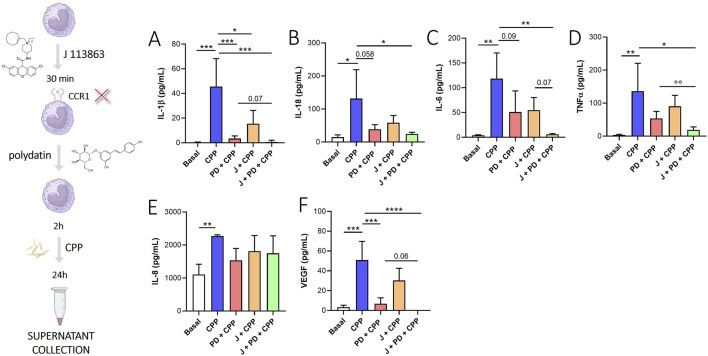
Effect of CCR1 inhibition on PD treatment. Monocytes were pretreated with PD (100 µM) for 2 h and then stimulated for 24 h with CPP (0.025 mg/mL). Where indicated, J113863 (10 µM) was added 30 min before the PD treatment. **(A)** IL-1β, **(B)** IL-18, **(C)** IL-6, **(D)** TNFα, **(E)** IL-8, **(F)** VEGF levels in supernatants were quantified by ELISA. Data are expressed as mean of four independent experiments ±SD. p calculated according to the One Way Anova, Bonferroni’s multiple comparisons test:*p < 0.05, **p < 0.01, ***p < 0.001, ****p < 0.0001. p calculated according to t-test, PD + CPP vs J + PD + CPP: °°p < 0.01. CPP, calcium pyrophosphate crystals; PD, polydatin.

We evaluated the effect of CCL-23 receptor (CCR1) inhibition through the use of J-1113863, a potent and selective CCR1 antagonist (Figure 5). J-1113863 treatment reduces IL-1β (Figure 5A, p < 0.05) release from monocytes stimulated with CPP crystals for 24 h. Although not significantly, we also observed a decrease of IL-6 (Figure 5C) and VEGF (Figure 5E) in monocytes treated with J-111386 versus monocytes stimulated with CPP crystals. However, we did not see any effects on the IL-8 and TNFα reduction following the use of J-1113863 (Figures 5C, D). The combined treatment with PD and J significantly reduced IL-1β (p < 0.001), IL-18 (p < 0.05), IL-6 (p < 0.01), TNFα (p < 0.05) and VEGF (p < 0.0001) levels compared to monocytes stimulated with CPP crystals. Intragroup differences showed that treatment with J111386 + PD blocks the release of TNFα (p = 0.01) more than PD treatment alone. Although not significant, this trend was also observed for IL-1β (p = 0.07), IL-6 (p = 0.07) and VEGF (p = 0.06) suggesting that PD, in addition to modulate CCL-23 signal, acts also through other mechanisms of action.

CCL-23 appears to be linked to the signal mediated by IL-1β and, furthermore, the inhibition of its receptor (CCR1) acts to modulate the IL-1β, IL-6 and VEGF release. Together, these results highlight the importance of this chemokine in CPP-induced inflammation and suggest that CCR1 modulation may become a new therapeutic strategy to modulate the release of the previously described mediators.

### 3.5 PD acts through SIRT1 modulation

**FIGURE 6:**
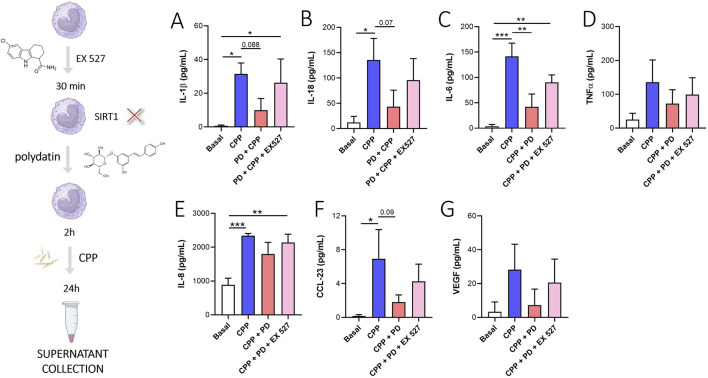
PD acts through SIRT1 modulation. Monocytes were pretreated with PD (100 µM) for 2 h and then stimulated for 24 h with CPP (0.025 mg/mL). Where indicated, EX527 (10 µM) was added 30 min before the PD treatment. **(A)** IL-1β, **(B)** IL-18, **(C)** IL-6, **(D)** TNFα, **(E)** IL-8, **(F)** CCL-23 and **(G)** VEGF levels in supernatants were quantified by ELISA. Data are expressed as mean of four independent experiments ±SD. p calculated according to the One Way Anova, Bonferroni’s multiple comparisons test: *p < 0.05, **p < 0.01, ***p < 0.001. CPP, calcium pyrophosphate crystals; PD, polydatin.

Among the mechanisms of action described in the literature, the PD action on the modulation of SIRT-1 is reported (Sun et al., 2021). In our *in vitro* experimental model, the inhibition of SIRT1 through EX527, a selective SIRT1 inhibitor, reversed the PD-mediated reduction of IL-1β (Figure 6A), IL-18 (Figure 6B), IL-6 (Figure 6C), IL-8 (Figure 6E) and CCL-23 (Figure 6F). However, the inhibition of SIRT1 did not affect significantly the release of TNFα (Figure 6D) and VEGF (Figure 6G).

### 3.6 PD and J113863 inhibit PBMCs chemotaxis

**FIGURE 7:**
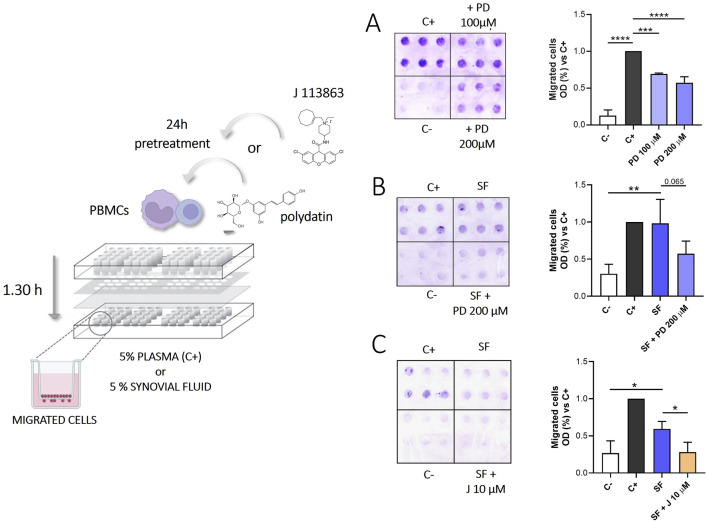
Migration of PBMCs exposed to SFs from patients with CPP induced arthritis in the presence or absence of PD (100–200 µM) or J113866 (10 µM) for 1.30 h. Medium RMPI supplemented with 5% plasma was used as a positive control (C+), while medium RPMI supplemented with 1% FBS was used as a negative control (C-). *Left panel*: Representative image of migrated cells on the bottom of a filter membrane of a modified 48-well Boyden chamber. *Right panel*: Effect of 100–200 μM PD on PBMCs migration induced by **(A)** 5% plasma (n = 3) **(B)** inflammatory SFs from CIA patients (n = 4). **(C)** Effect of 10 μM J113863 on PBMCs migration induced by inflammatory SFs from CIA patients (n = 4). Cell migration is shown as optical density values (O.D). Each sample was tested in sextuplicate. Data are expressed as mean of three to four independent experiments ±SD. p calculated according to the One Way Anova, Bonferroni’s multiple comparisons test: *p < 0.05, **p < 0.01, ***p < 0.001, ****p < 0.0001. CPP, calcium pyrophosphate crystals; PD, polydatin; SF, synovial fluid; PBMCs, peripheral blood mononuclear cells.

We investigated the effect of PD on the migration of PBMC, using plasma and SF from CIA patients as chemoattractant stimuli. As shown in Figures 7A, B, treatment with 200 µM PD reduced PBMCs migration induced by plasma (p < 0.0001) and, even if the difference is not significant, it decreases migration induced by SFs (p = 0.065).

Recently data from the literature shown that CCL-23 induces the migration of monocytes (Kim et al., 2011) and CCR1 blockade ameliorates the development of CIA in an *in vivo* model, probably via inhibition of inflammatory cell recruitment (Amat et al., 2006a). Therefore, we performed additional experiments to test the effect of CCR1 inhibition on PBMCs migration in response to SFs. As shown in Figure 7C, J-113863 inhibited the migration of PBMCs induced by SFs (p < 0.05). These results suggested that CCR1 was involved not only in cell activation but also in chemotaxis.

## 4 Discussion

The anti-inflammatory properties of PD have been observed in different inflammatory experimental models of disease (Tang et al., 2019; Lv et al., 2018; Yang et al., 2019; Ravagnan et al., 2013). Recently, we evaluated the role of this compound in CPP-induced inflammation both *in vitro* and *in vivo* experiments. In our models, PD has shown to prevent the development of CPP crystal-induced acute arthritis in mice (Oliviero et al., 2021) and suppress the inflammatory response triggered by crystals in THP-1 cells (Oliviero et al., 2019).

With this work, we sought to investigate specific mechanisms of action of PD showing a multitargeted biological properties in preventing acute pseudogout attacks in mice. Considering our previous *in vivo* results, we first used a pilot experiment in mice to confirm PD’s preventive effect in acute arthritis and to perform a preliminary protein array to guide subsequent investigations. Additionally, we also pursued to examine the effect of CPP crystals and PD in skeletal muscles. We confirmed that PD, administered prophylactically, prevented ankle swelling, leukocyte infiltration, edema, and synovitis 48 h after crystal injection (Figure 1). These effects were similar to those obtained with colchicine, the most common drug used to treat acute episodes of crystal-induced arthritis. Furthermore, PD treatment reduced the inflammatory damage of muscles included leukocyte infiltration, edema and reverses muscle weakness induced by CPP injection (Figure 2). These findings suggest that PD could provide a potential benefit in preventing the development of disease flares.

However, until now, the mechanisms underlying PD’s protective effects in CPP-induced arthritis are not fully understood.

To address this, we performed a pilot protein array analysis of joint tissues to evaluate the main crystal-activated pathways potentially targeted by PD. Our results highlighted that CPP crystals in mice increased the levels of several factors involved in the leukocytes and endothelial cell migration (MIP-1γ, VCAM, L-selectin, BLC), angiogenesis (VEGF, VEGF-R) and inflammation (Supplementary Figure S1).

To validate these preliminary findings, we used an *in vitro* model of CPP-induced inflammation. Monocytes from HDs were pretreated with PD for 2 h and then stimulated with CPP crystals for 24 h. Our *in vitro* results showed that CPP crystals induced the release of pro-inflammatory cytokines (IL-1β, IL-18, IL-6, TNFα), chemokines (CCL-23, IL-8) and VEGF after 24 h. PD pretreatment reduced the release of all these inflammatory mediators and VEGF (Figure 3).

IL-1β reduction may be in line with results by Chen et al., who demonstrated that a PD-mediated downregulation of NF-κB, NLRP3, ASC and caspase-1 expression leads to decresed IL-1β secretion in potassium oxonate-induced hyperuricemic rats (Chen and Lan, 2017).

Notably, our study identified CCL-23, a chemokine not previously reported in CPP-induced inflammation, as a key mediator. Known as myeloid progenitor inhibitory factor (MPIF)-1, CCL-23 acts through the CCR1 receptor and has properties similar to those of mouse MIP-1γ. The expression of CCL-23 mRNA was found in monocytes stimulated with IL-1β (Forssmann et al., 1997) and IL-4 (Nardelli et al., 1999; Novak et al., 2007) and can also be detected in SFs from patients with rheumatoid arthritis (Berahovich et al., 2005). In inflammatory diseases, this chemokine acts as chemoattractants for immune effector cells when it binds to its receptor (Patel et al., 1997; Youn et al., 1998) and, is also involved in VEGF-induced propagation and migration of endothelial cells and MMP-2 secretion (Hwang et al., 2005; Han et al., 2009; Son et al., 2006). Beyond its efficacy in reducing the release of pro-inflammatory cytokines and chemokines (Tang et al., 2018; Guang et al., 2017; Wu et al., 2020), PD also exerts an inhibitory effect on VEGF levels, which was particularly intriguing.

PD action on this growth factor was recently described by Hu et al. that showed PD inhibition of angiogenesis by its binding to VEGF (Hu et al., 2019). Here, we showed that PD acts also by preventing VEGF release from monocytes stimulated with CPP crystals, suggesting that PD may have a role in modulating endothelial permeabilization and migration.

Interestingly, IL-1β signaling inhibition using anakinra also reduced IL-18, IL-6, TNFα, IL-8, CCL-23 and VEGF levels. The efficacy of anakinra in CCL-23 inhibition was interesting, and there is no evidence from the literature that connects IL-1β signaling with CCL-23. PD cotreatment with anakinra appears to have a combined effect in reducing inflammatory mediators after CPP crystals stimulation compared to anakinra or PD treatment alone, suggesting that PD does not act only through IL-1β reduction (Figure 4).

To further explore PD’s mechanisms of action, we investigated the role of CCR1, a receptor for CCL-23, using the selective antagonist J-113863. Previously published studies reported that CCR1 inhibition was effective in reducing collagen induced arthritis in mice and alters cytokine networks (Amat et al., 2006b), in decreasing inflammation in mice with glomerulosclerosis and nephrotic syndrome (Vielhauer et al., 2004) in suppressing pro-inflammatory expression and up-regulating anti-inflammatory mediators in a mouse model of relapsing-remitting multiple sclerosis (Ansari et al., 2022).

Surprisingly, in our experimental model, CCR1 inhibition significantly reduced the release of IL-1β, IL-18, IL-6, TNFα and VEGF (Figure 5), highlighting its involvement in the inflammatory response. Notably, combined PD and J-113863 treatment demonstrated enhanced efficacy in suppressing inflammatory mediators compared either treatment alone.

Among the mechanisms of action proposed in the literature, SIRT-1 activation by PD has been reported (Sun et al., 2021). However, no evidence was found regarding CPP crystal-induced inflammation. In our experimental model, inhibition of SIRT-1 through EX527, reverts PD-mediated reduction of IL-1β, IL-18, IL-6, IL-8, CCL-23 and VEGF (Figure 6).

We also evaluated the role of PD in preventing leukocytes migration. Cheng Y et al. found that PD exerted protective effects through a decrease in the expression of ICAM-1 and VCAM-1 in brain tissues from ischemia-reperfusion injury (Cheng et al., 2006). Therefore, we investigated the effect of PD pretreatment in PBMCs migration through a microchemotaxis chamber in response to plasma and SFs from patients with CPP crystal-induced arthritis. SFs from these patients can be considered representative of the inflammatory environment that is generated following the acute attack. We previously reported that SFs contain high levels of cytokine, chemokines and growth factors (Baggio et al., 2023) that can induce chemotactic migration of leukocytes.

Migration inhibition was observed in response to all stimuli tested (Figures 7A, B). These data suggest that PD may prevent inflammatory damage by reducing leukocyte chemotaxis, consistent with our *in vivo* results, where PD pretreatment led to the downregulation of VCAM, L-selectin, and BLC (Supplementary Figure S1).

Finally, we tested the effect of CCR1 inhibition on PBMCs migration in response to SFs (Figure 7C). It is reported that CCR1 expression is associated to migration of leukocytes and its wide cellular distribution, together with its role in both cell migration and activation, suggests that it may have a pleiotropic role in CPP mediated inflammation (Nardelli et al., 1999; Patel et al., 1997; Youn et al., 1998). Furthermore, we found high levels of CCL-23 in SFs (663 pg/mL, IQR: 404–811) that can act as chemoattractant stimuli trough CCR1 stimulation. CCR1 inhibition effectively reduced PBMCs migration in response to SFs.

Collectively, our results showed that CPP crystal-driven inflammatory response is mediated by a complex network of pro-inflammatory mediators, with chemokines playing a pivotal role. Dysregulation of this process might cause the sustained leukocyte recruitment. In this perspective, PD and CCR1 blockade seems to be effective in reducing leukocyte migration and preventing inflammatory damage.

Overall, our results showed that PD pretreatment reduced joint and muscle inflammatory damage. PD acts through different mechanisms consisting in reducing inflammatory cytokines and inhibiting leukocyte migration probably also through the reduction of chemokines such as CCL-23. Our data showing reduced VEGF and CCL-23 production in PD-treated monocytes are interesting to further investigate the antiangiogenic potential of PD in endothelial cells. Future studies will focus on evaluating the impact of PD on CCR1 expression and its ligands.

## Data Availability

The raw data supporting the conclusions of this article will be made available by the authors, without undue reservation.
