# Supplementary material for: Multitargeted biological actions of polydatin in preventing pseudogout acute attack
Source: Front Mol Biosci. 2025 Feb 27;12:1553912. doi: 10.3389/fmolb.2025.1553912 (PMC11903430; doi:10.3389/fmolb.2025.1553912)
Supplement: Supplementary file 1 [file DataSheet1.docx]

Supplementary Material

Supplementary Table 1. Characteristics of synovial fluids included in the study

| **WBC, n/mm^3^ (IQR)** | 23000 (8900 - 47600) |
| --- | --- |
| **PMN, % (IQR)** | 89 (81 - 96) |
| **M, % (IQR)** | 10 (4 - 18) |
| **L, % (IQR)** | 1 (0 - 2) |
| **CCL-23, pg/mL, (IQR)** | 663 (404 - 811) |

SF was examined as part of a routine procedure including ordinary light microscopy examination for total and differential white blood cell (WBC) count using a Bürker counting chamber and pre-stained slides for cell morphology (Testsimplets®), respectively. Differential WBC provided the percentage of polymorphonuclear (PMN), monocytes (M) and lymphocytes (L) in the SF. Data are expressed as the median and interquartile range (IQR). WBC, white blood cell; PMN, polymorphonuclear cells; M, monocytes; L, lymphocytes; IQR, interquartile range.

# Supplementary Figure 1.
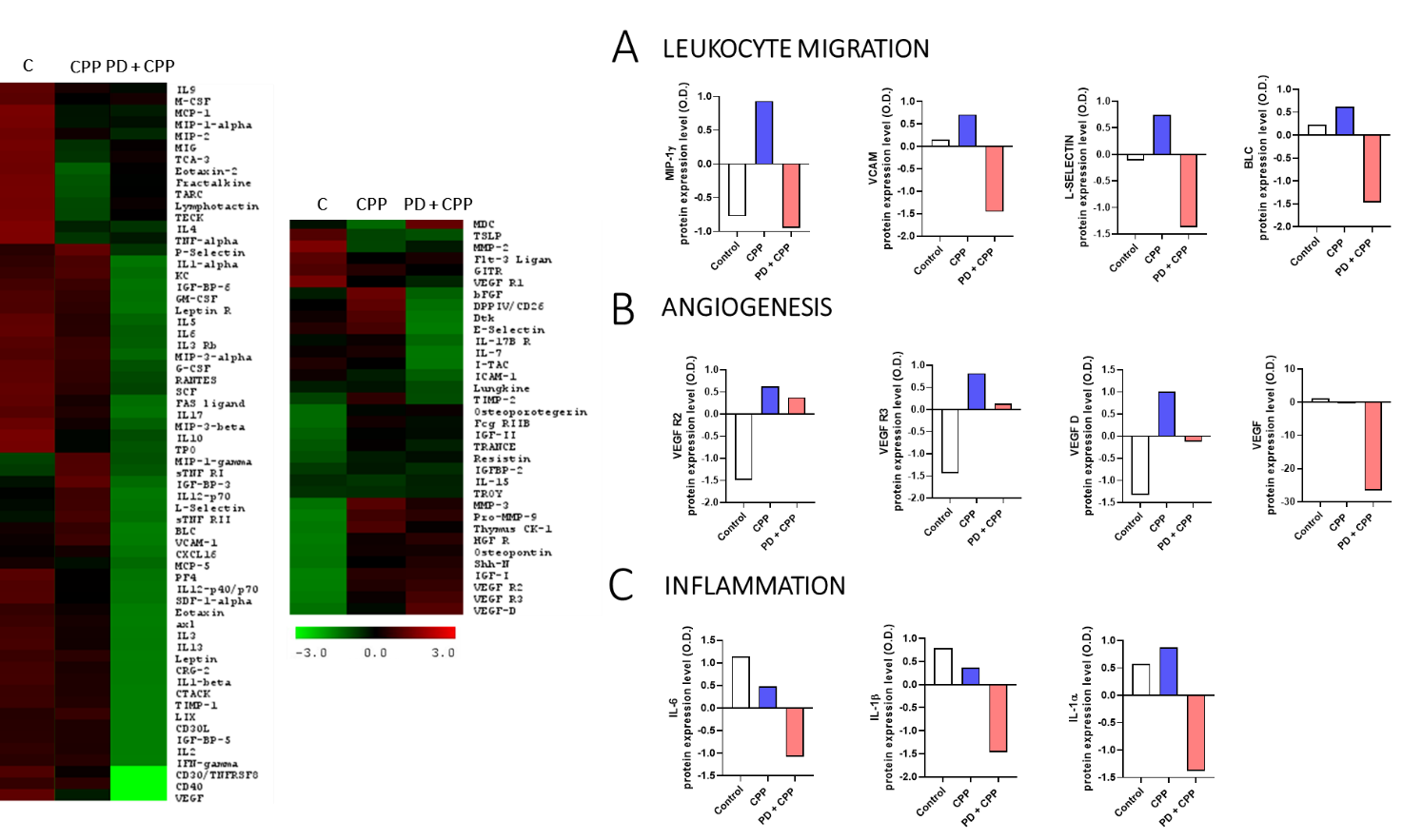


**Supplementary Figure 1. Protein Array.** Protein array was performed using pooled lysates from mice (n = 2) ankles as described in Materials and Methods. *Left panel*: Heatmap of the proteins involved in leukocytes migration, angiogenesis and inflammation in ankles of mice treated with CPP and CPP + PD, and control. Red and green colors indicate high and low protein expression, respectively. *Right panel*: (**A**) Upregulated expression levels of proteins associated with leukocytes migration: MIP-1γ, VCAM, L-SELECTIN, BLC. Results are reported as optical density (OD) arbitrary units. (**B**) Upregulated expression levels of proteins associated with angiogenesis: VEGF-R2, VEGF-R3, VEGF D, VEGF. Results are reported as optical density (OD) arbitrary units. (**C**) Upregulated expression levels of inflammatory mediators: IL-6, IL-1β, IL-1α. Results are reported as optical density (OD) arbitrary units. CPP, calcium pyrophosphate crystals; PD, polydatin.
